# Séroprévalence de l'hépatite virale C à l'Hôpital Militaire d'Instruction Mohammed V de Rabat

**DOI:** 10.11604/pamj.2014.19.182.5347

**Published:** 2014-10-21

**Authors:** Taoufik Doblali, Rachid Hadef

**Affiliations:** 1 Pôle Biologie l'Hôpital Militaire d'Instruction Mohammed V CP: 10000 Rabat Hay Ryad, Maroc; 2 Centre de Transfusion Sanguine Hôpital Militaire d'Instruction Mohammed V CP: 10000 Rabat Hay Ryad, Maroc

**Keywords:** Hépatite C virale, hémodialysés chronique, donneurs sang, séroprévalence, Hepatitis C, chronic hemodialysis, blood donor, seroprevalence

## Abstract

**Introduction:**

L'hépatite virale C représente un problème majeur de santé publique. L'OMS estime qu'il y'aurait 180 millions de porteurs chroniques de part le monde. L'objectif de cette étude est de connaître la séroprévalence de l'hépatite C virale à l'hôpital militaire d'instruction Mohammed V de Rabat.

**Méthodes:**

À travers cette étude rétrospective, menée entre janvier 2010 et décembre 2012 et qui Concerne les patients hémodialysés chroniques du centre d'hémodialyse (groupe 1, n =152), les donneurs de sang du centre de transfusion (groupe 2, n= 29530) et les jeunes recrues des forces armées royales marocaine en visite d'aptitude (groupe 3, n = 15320). Au total 45002 sérums ont été testés par technique immunoenzymatique au laboratoire de l'HMIMV.

**Résultats:**

La séroprévalence du virus de l'hépatite C est respectivement de 17,8% pour le groupe1, de 0,18% pour le groupe 2 et de 0,25% pour le groupe 3.

**Conclusion:**

La faible prévalence de l'hépatite C enregistrée dans notre étude semble en rapport avec leur faible endémicité au Maroc. Les taux de prévalence retrouvés sont inférieurs à ceux enregistrés dans la population générale traduisant l'efficacité des programmes de surveillance, de prévention et de prise en charge des infections hépatiques adoptés au sein du Service de santé des forces armées royales marocaines.

## Introduction

L'hépatite virale C (VHC) représente un problème majeur de santé publique. L'OMS estime qu'il y aurait 180 millions de porteurs chroniques de part le monde [1]. La systématisation du dépistage du VHC sur les dons de sang, lors des recrutements, et dans le suivi des hémodialysés chroniques constitue un des principaux moyens de lutte et de prévention de sa dissémination. Dans notre étude, nous déterminons la séroprévalence du VHC à l'hôpital militaire d'instruction Mohammed V (HMIMV) de Rabat chez trois groupes de patient: le groupe 1: hémodialysés chroniques (HDC)( n:152, âge compris entre 35 et 70 ans) le groupe 2: donneurs de sang (n: 29530, âge compris entre 18 et 45 ans) et le groupe 3: jeunes recrues des forces armées royales (n: 15320, âge compris entre 17 et 22 ans) et nous comparons nos données à ceux de la littérature.

## Méthodes

Il s'agit d'une étude rétrospective menée du 1^er^ janvier 2010 au 31 décembre 2012. Au total 45002 sérums ont été testés par technique immunoenzymatique de type ELISA (enzyme linked immunosorbent assay) de 4^ème^ génération (kit Monolisa HCV Ag-Ab^R^ de BioRad). Les différents tests ont été pratiqués suivant les recommandations et protocoles des fournisseurs. Tout test positif ou douteux (zone grise en ELISA) a fait l'objet d'un contrôle en double avec le même réactif ou un autre réactif et le sujet faisait l'objet d'une prise en charge spécifique (information, test de confirmation). Les tests de confirmation pour le VHC sont exécutés par le service de virologie du même établissement par technique ECLIA (Electro- chemi luminescence Immuno-assay) sur cobas E 411 (kit anti HCV II roche) La répétabilité ou la discordance des résultats en ELISA entraînait la disqualification immédiate du don de sang, l'isolement technique de l'HDC et l'inaptitude de la jeune recrue (après confirmation).

## Résultats

La séroprévalence du VHC est respectivement de 17,8% pour le groupe 1, de 0,18% pour le groupe 2 et de 0,25% pour le groupe 3 (Tableau 1).

**Tableau 1:** Récapitulatifs des résultats

Groupe	Nombre de patient	Nombre de cas positifs	Prévalence
1	152	27	17,8%
2	29530	53	0,18%
3	15320	38	0,25%

## Discussion

La séroprévalence du VHC est beaucoup plus élevée en dialyse que dans la population générale ou elle ne dépasse pas 20% dans les pays endémiques. Chez les HDC, cette prévalence, comme celle de la population générale, varie selon les zones géographiques et selon les pays entre 2% au Royaume-Uni et 20% en Italie. Dans les pays en développement, la situation est particulière car la prévalence du VHC peut dépasser les 80% [2].

Au Maroc, cette séroprévalence est de 32% selon le registre national Maroc-greffe dialyse (Magredial) [2]. Cependant, il s'agit d'une moyenne globale car la prévalence varie, dans le même pays, selon les unités de dialyse. Dans notre étude, la séroprévalence du VHC est de 17,8%. Elle est plus basse que celle décrite par le Magredial, mais plus élevée que dans la population générale du Maroc, qui figure dans les pays d'endémicité moyenne (prévalence de l'ordre de 1%) [1], et que dans la population des donneurs de sang 1,08% [2, 3]. Les principaux facteurs de risque chez l'HDC sont l'ancienneté de la dialyse et la transfusion sanguine, qui ont perdu de leur importance grâce au dépistage systématique des anticorps anti-VHC instauré en 1994 chez les donneurs de sang ainsi qu'un dépistage régulier des patients séropositifs par la recherche des anticorps anti-VHC et surtout de l'ARN viral instauré chez les HDC de l'HMIMV depuis avril 2010 selon les recommandation du KDIGO (Kidney Disease Improving Global Outcomes) dans les situations de haute prévalence[2]. La transmission nosocomiale est certaine dans les services d'hémodialyse, nécessitant une meilleure observance des règles d'hygiène universelles. Un contrôle régulier et systématique du respect de ces mesures d'hygiène doit être institué dans les centres d'hémodialyse [2].

Les jeunes recrues des forces armées royales et les donneurs de sang de notre étude sont des militaires expliquant les caractéristiques socio démographique de notre échantillon: population jeune et masculine à 98% Dans notre étude, la séroprévalence du VHC s'avère nettement plus faibles que celle de la population générale et des donneurs de sang avec 0,25% pour les jeunes recrues et 0,18% pour les donneurs de sang. Elles se rapprochent de celles rapportées dans une population de donneurs de sang en Tunisie [3] et diffèrent de celles élevées des régions géographiques de forte endémicité [3]. Nos données comparées à celles obtenues par deux études antérieurement menées dans le service de transfusion sanguine de l'HMIMV montrent une nette tendance à la baisse des taux de séroprévalence mesurés passant de 0,30% entre 1991 et 1997 à 0,18% actuellement traduisant une amélioration significative de la sélection des donneurs [3]. Nos séroprévalences sont aussi plus basses que celle rapportées lors d'une étude à l'hôpital militaire Moulay Ismael de Meknes qui étaient de 0,35% pour les jeunes recrues et de 0,33% pour les donneurs de sang [4].

## Conclusion

La faible séroprévalence du VHC enregistrée dans notre étude semble en rapport avec sa faible endémicité au Maroc. Les taux de prévalence retrouvés sont inférieurs à ceux enregistrés dans la population générale traduisant l'efficacité des programmes de surveillance, de prévention et de prise en charge adoptés au sein du Service de santé des forces armées royales marocaines. Toutefois, la sécurité transfusionnelle pour tout don, le respect des règles d'hygiènes universelles et le suivi sérologique des HDC ainsi que le dépistage systématique des jeunes recrues, visent à réduire au maximum, mais sans éliminer le risque viral résiduel que représente la fenêtre virologique du virus de l'hépatite C, qui pourra être réellement réduit avec l'instauration systématique de la recherche de l'ARN viral surtout pour la qualification des dons de sang.
